# Orthodontics in the oral health care network of the Unified Health System (SUS)

**DOI:** 10.1590/1807-3107bor-2024.vol38.0011

**Published:** 2024-01-05

**Authors:** Fábio Carneiro MARTINS, Brunna Rodrigues Machado dos SANTOS, Edgard Michel CROSATO, Maria Clara Lembro TEIXEIRA, Mariana GABRIEL, Maria Ercília de ARAÚJO, Paulo Savio Angeiras de GOES, Fernanda Campos de Almeida CARRER

**Affiliations:** (a) Universidade de São Paulo – USP, School of Dentistry, Department of Community Dentistry, São Paulo, SP, Brazil.; (b) Universidade de Mogi das Cruzes - UMC, School of Dentistry, Department of Community Dentistry, Mogi das Cruzes, SP, Brazil.; (c) Universidade Federal de Pernambuco – UFPE, School of Dentistry, Department of Clinical and Preventive Dentistry, Recife, PE, Brazil.

**Keywords:** Orthodontics, Oral Health, Health Policy, Comprehensive Health Care

## Abstract

This observational study aimed to describe and analyze data from two external evaluations of the National Program for Improving Access to and Quality of Dental Specialty Centers (PMAQ CEO), held in 2014 and 2018 in Brazil, which evaluated Dental Specialty Centers (CEO) using a national and census approach. We selected questions through a search in the microdata of the first and second evaluations. The groups were analyzed independently. To compare the groups, nonparametric tests were performed (Mann Whitney U). The formulated hypotheses were: there would be no differences between the data of these groups (h0) and there would be differences between the data of these groups (h1). For qualitative nominal variables, frequency distribution was verified and association tests were performed (chi-square test). The significance level for this study was set at 5%. We observed that orthodontic treatments were found in about 13% of the CEO. Regarding human resources, most professionals were specialists or had MSc or PhD degrees; were civil servants; had been hired by direct administration; or had been hired via public tender. Regarding the work process and inclusion of the CEO in the health care network, we observed a greater number of services that use single and electronic medical records, greater presence of services monitoring and analyzing goals, greater knowledge about monthly average of absenteeism (for 2018); and larger number of services with referrals from primary health care centers (for 2014). Expanding the view on orthodontics and including preventive, interceptive, and corrective treatments at different points in health care networks are essential strategies for achieving comprehensive care in universal health systems.

## Introduction

Malocclusion is defined as deviation of dental arches or facial skeleton, or both, from the normal pattern, with impaired function of the stomatognathic system and impact on the appearance and self-esteem of those affected.^1,2^ It has the third highest prevalence rate among oral health problems according to the World Health Organization (WHO), outranked only by dental caries and periodontal diseases.^3^ In addition, malocclusion is also related to the risk of dental trauma, increasing the morbidity associated with the loss of tooth elements associated with these risks.^4^ The occurrence of malocclusion has been associated with factors such as social inequalities, behavior, genetics, and other oral diseases such as dental caries, which can cause the early loss of deciduous and permanent teeth, with consequent migration of other tooth elements to the prosthetic spaces created by these losses.^5^

In Brazil, a National Oral Health Survey was carried out in state capitals and in towns in the interior of the five Brazilian regions in 2010 (“SB Brasil 2010”), using multistage cluster sampling, with census sector and domicile as sample units. Thirty sectors in the state capitals and 30 municipalities within each region were drawn. Accuracy considered the domains grouped according to the degree of overall population density and the internal variability of the indices. Dental caries, periodontal disease, malocclusion, fluorosis, dental trauma, and edentulism were evaluated in five age groups (5; 12; 15 to 19; 35 to 44; and 65 to 74 years), with the objective of providing the Brazilian Ministry of Health and institutions affiliated with the Unified Health System (SUS) with information for the development of programs in the sector.^6^ In terms of general organization, SB Brasil 2010 is a national survey, including state capitals, the Federal District, and the five macroregions (North, Northeast, Southeast, South, and Midwest).

According to this survey, 38.8% of 12-year-old adolescents had malocclusion.^7^ The impact of this problem on the perception of appearance can affect mental health and social behavior, with significant implications for areas of social interaction, such as education, work, and emotional life.^1,2^ This impact occurs mainly in cases of disharmonious facial asymmetries and is even greater in more vulnerable populations because of other social determinants,^8,9^ posing a huge challenge. Nowadays, Brazil has the largest number of specialists in orthodontics (28,240 orthodontists registered with the Brazilian Federal Council of Dentistry – CFO – in 2020) – 23.1% accounted for professionals who have attended a certificate program and 8.2% for the total of dental surgeons in Brazil.^10^

The Brazilian oral health care has been remodeled since 2004, with the implementation of the National Oral Health Policy known as Smiling Brazil (BS). The federal government assumed an important role in increasing the offer of oral health services in municipalities and states, creating specific financing lines for the new Oral Health Teams (OHTs), construction and implementation of secondary care centers (Dental Specialty Centers – CEO), tertiary care centers, among other actions, which made BS a health care network and one of the largest public oral health policies in the world.^11^

The implementation of CEO (Ordinance no. 599 of 2006) was one of the major milestones of the BS program.^12^ Periodontics, surgery, endodontics, prosthodontics, and care for patients with special needs are offered by all CEO, with the possibility of including other specialties. Orthodontic and implant treatments were the last to be included, and the possibility of financing by the Brazilian Ministry of Health (Ministerial Ordinance No. 718 / SAS of 12/20/2010) was opened.

The reasons for carrying out this study were the high prevalence of malocclusion, the large number of orthodontists in Brazil, and the possibility of working with the Unified Health System. This study aimed to identify, compare data from the two evaluations performed at all CEO in Brazil, related to orthodontics in Brazilian public health services, and verify the possibilities that orthodontics offers to universal health systems. It was based on the indicators of the National Program for Improving Access to and Quality of Dental Specialty Centers, the biggest institutional evaluation program in the history of Smiling Brazil (known as PMAQ-CEO, this program was established within the scope of the National Oral Health Policy through Ordinance GM/MS no. 261, of February 21, 2013, and had its rules revised by Ordinance GM/MS no. 1599, of September 30, 2015). It resulted from an important negotiation and agreement established by the three spheres of SUS management, with the objective of promoting more access to CEO and improving the quality of their services, with the guarantee of nationally, regionally, and locally comparable quality standards in order to allow greater transparency and effectiveness of government actions).^13^

## Methods

This is an observational study that used secondary data from the first and second cycles of the PMAQ – CEO, performed in 2014 and 2018, respectively. Through the program, CEO were certified according to their performance, including self-evaluation by CEO professionals, evaluation of performance of the set of contractual indicators, and evaluation of external performance. For this study, we used data from the external performance evaluation. This phase was divided into three modules: a) direct observation, b) interview with CEO manager, c) interviews with professionals and service users. The information was locally recorded by previously selected and trained dental surgeons, with a national and census approach.^14^ Data were collected using a tablet and then immediately sent to the Brazilian Ministry of Health after submission of the evaluation.

For this study, we selected questions of the instrument used for the external evaluation of CEO (AE-PMAQ-CEO), obtained from an active search in the microdata of the external evaluation of the first cycle of PMAQ – CEO,^15^ and in the microdata of the second cycle (available at: https://aps.saude.gov.br/ape/pmaq/ciclo2ceo/). The questions related to orthodontics used in this study were categorized, as shown in Figure. All the questions selected from the microdata were restricted to CEO with orthodontic services. These variables are characteristic of CEOs that offer orthodontic treatment; therefore, they represent all the specialties of these CEO, and not just orthodontics.

**Figure 1:**
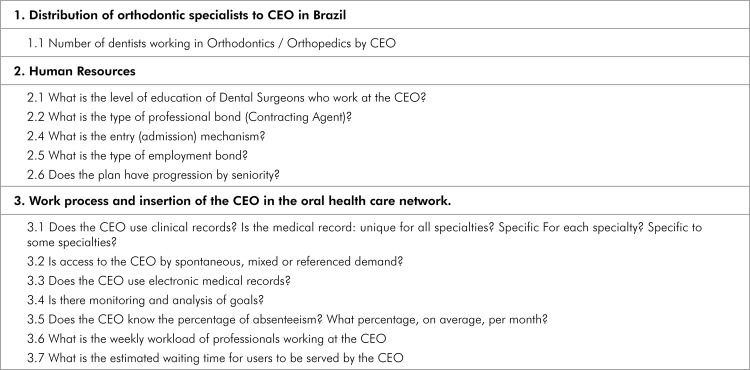
Questions of the microbank data related to CEO with orthodontics

The data were tabulated into Excel spreadsheets and categorized according to their characteristics into quantitative or qualitative nominal variables. The evaluated CEO were divided into two groups: a) PMAQ-CEO 1 – evaluated in the first cycle of the AE-PMAQ-CEO – 2014; b) PMAQ-CEO 2 – evaluated in the second cycle of the AE-PMAQ-CEO – 2018) and analyzed as independent groups, using Jamovi 1.2.26 statistical software.^16^

For quantitative data, the distribution of the sample data was verified by the Shapiro-Wilk normality test. To compare the groups, nonparametric tests were performed (Mann Whitney U), and the formulated hypotheses were: there would be no differences between the data of these groups (h0) and there would be differences between the data of these groups (h1), i.e., there would be differences between the data for CEO that offered orthodontics in the 2014 and 2018 external evaluations. For qualitative nominal variables, frequency distribution was verified, and association tests were performed (chi-square test). The significance level for this study was set at 5% for all tests performed.

This study used secondary data, and to collect data using the PMAQ-CEO external evaluation, the research was approved by the Research Ethics Committee of the Universidade Federal de Pernambuco (UFPE), process no. 2.478.524 and CAAE 23458213.0.0000.5208.

## Results

The data were presented according to the categories described in Figure (distribution of orthodontic specialists at CEO in Brazil, human resources, work process, and inclusion of CEO in the oral health care network).

As described in Table 1, it was found that orthodontics was present in less than 15% of CEO in Brazil in the two AE-PMAQ-CEO. The Midwest region has the lowest number of services with orthodontic treatment, failing to reach 6% in either of the two PMAQ-CEO cycles. The Southeast and Northeast regions concentrate about 80% of the CEO that offer orthodontic treatments.

**Table 1:** CEOs with orthodontic treatment in Brazil and their geographic distribution: frequency distribution measures

A - Number of CEO with orthodontic treatment in Brazil	Total number of CEO evaluated	Total number of CEO with orthodontic treatment (%)
PMAQ-CEO I	934	124 (13.2)
PMAQ-CEO II	1097	139 (12.7)
B - Geographic distribution (by macroregion)	n (%)	n (%)
North	PMAQ-CEO 1	8 (6.4)
	PMAQ-CEO 2	8 (8.6)
Northeast	PMAQ-CEO 1	43 (34.7)
	PMAQ-CEO 2	45 (32.4)
Southeast	PMAQ-CEO 1	55 (44.4)
	PMAQ-CEO 2	62 (44.6)
South	PMAQ-CEO 1	12 (9.7)
	PMAQ-CEO 2	12 (8.6)
Midwest	PMAQ-CEO 1	6 (4.8)
	PMAQ-CEO 2	8 (5.7)
Total	PMAQ-CEO 1	124 (100)
	PMAQ-CEO 2	139 (100)

Data related to human resources are presented in Table 2 according to the measures of central tendency and dispersion. For all variables studied, there was no parametric distribution of data according to normality tests. The p value of the nonparametric comparison tests is also shown in Table 2. We noticed that between 2014 and 2018, there was a small decrease in the average number of professionals who only have undergraduate degrees or refresher courses, a decrease in the average of those hired by other agents (not by the direct administration), and a decrease in the average number of professionals who were not hired via public competitive examination or public selection process.

**Table 2:** Human resources (central tendency and dispersion measures; nonparametric comparison tests – Mann Whitney.

Variable	n	Mean (SD)	Median (IQ range)	p-value
A. Professional qualification				0.051**
College degree only				
PMAQ-CEO 1	124	0.16 (1.02)	0.00 (0–0)	
PMAQ-CEO 2	138	0.03 (0.21)	0.00 (0–0)	
Refresher courses				
PMAQ-CEO 1	124	0.13 (0.38)	0.00 (0–0)	0.033*
PMAQ-CEO 2	138	0.04 (0.21)	0.00 (0–0)	
Certificate program /Master’s / PhD degree				
PMAQ-CEO 1	123	1.93(1.50)	1.00 (1–3)	0.193
PMAQ-CEO 2	138	1.75(1.57)	1.00 (1–2)	
B. Contracting agent				
Direct administration				
PMAQ-CEO 1	124	9.35 (8.29)	8.00 (2–14)	0.976
PMAQ-CEO 2	138	9.90 (9.78)	8.00 (0.25–15)	
Others				
PMAQ-CEO 1	124	4.12 (6.70)	0.00 (0–6.25)	0.004*
PMAQ-CEO 2	138	3.08 (6.58)	0.00 (0–0)	
C. Admission mechanism				
Public competitive examination				
PMAQ-CEO 1	124	8.53 (8.16)	6.50 (1–14)	0.564
PMAQ-CEO 2	138	9.25 (8.57)	8.00 (0–15)	
Public selection process				
PMAQ-CEO 1	124	3.33 (6.01)	0.00 (0–5)	0.144
PMAQ-CEO 2	138	4.04 (6.30)	0.00 (0–6)	
Others				
PMAQ-CEO 1	124	2.02 (4.56)	0.00 (0–2.25)	< 0.001*
PMAQ-CEO 2	138	0.67 (2.13)	0.00 (0–0)	
D. Employment bond				
Civil servant under a statutory contract				
PMAQ-CEO 1	124	7.82 (8.16)	5.50 (0–13)	0.684
PMAQ-CEO 2	138	7.92 (7.26)	7.50 (0–14)	
Commissioned position				
PMAQ-CEO 1	124	0.19 (0.85)	0.00 (0–0)	0.327
PMAQ-CEO 2	138	0.15 (0.85)	0.00 (0–0)	
Temporary employment contract				
PMAQ-CEO 1	124	1.56 (3.12)	0.00 (0–2)	0.708
PMAQ-CEO 2	138	1.55 (3.21)	0.00 (0–1)	
Employment contract (regulated by CLT)				
PMAQ-CEO 1	124	4.10 (6.77)	0.00 (0–6)	0.489
PMAQ-CEO 2	138	3.81 (6.45)	0.00 (0–4)	
Self-employed				
PMAQ-CEO 1	124	0.09 (0.77)	0.00 (0–0)	0.332
PMAQ-CEO 2	138	0.06 (0.68)	0.00 (0–0)	
Others				
PMAQ-CEO 1	124	0.31 (2.73)	0.00 (0–0)	0.506
PMAQ-CEO 2	138	0.16 (0.08)	0.00 (0–0)	

U*statistically significant; **at the limit of statistical significance.

Data related to the work process and inclusion of CEO in the oral health care network are presented in Table 3 according to the measures of central tendency, dispersion, and p value of the nonparametric comparison tests (for quantitative data); frequency distribution, and p value of the chi-squared tests (for qualitative data). As for the statistically significant results, there was an increase in the number of professionals who worked with a single medical record (the number of CEO which did not work with own medical records for each specialty increased from 77.4% to 97.8% between 2014 and 2018) and with electronic medical records (increase from 10.5% to 63.8%). In addition, there was an increase in dental care with mixed demand (spontaneous and by referral) from 37.1% to 62.3% and a consequent decrease in demand by referral only (from 62.1% to 37.7%). It is also noted that the monitoring and analysis of targets decreased (from 93.5% to 80.4%).

**Table 3:** Work process and inclusion of CEOs in the oral health care network.

Variables				
A. Qualitative variables	Yes	No		p-value
Type of medical record - One for all specialties				
PMAQ-CEO 1	74 (59.7)	50 (40.3)		0.008
PMAQ-CEO 2	102 (73.9)	34 (24.5)		
Specific for each specialty				
PMAQ-CEO 1	21 (16.9)	103 (83.1)		0.112
PMAQ-CEO 2	34 (24.6)	102 (73.4)		
Some specialties have their own medical records				
PMAQ-CEO 1	28 (22.6)	96 (77.4)		< 0.001*
PMAQ-CEO 2	0 (0.0)	136 (97.8)		
Access to the CEO is on demand: Spontaneous demand				
PMAQ-CEO 1	1 (0.8)	123 (99.2)		0.291
PMAQ-CEO 2	0 (0.0)	138 (100)		
Mixed demand (spontaneous and by referral)				
PMAQ-CEO 1	46 (37.1)	78 (62.9)		< 0.001*
PMAQ-CEO 2	86 (62.3)	52 (37.4)		
Demand by referral				
PMAQ-CEO 1	77 (62.1)	47 (37.9)		< 0.001*
PMAQ-CEO 2	52 (37.7)	86 (61.8)		
Does the CEO use electronic medical records?				
PMAQ-CEO 1	13 (10.5)	111 (89.5)		< 0.001*
PMAQ-CEO 2	88 (63.8)	49 (35.5)		
Is there monitoring and analysis of goals?				
PMAQ-CEO 1	116 (93.5)	8 (6.5)		0.002*
PMAQ-CEO 2	111 (80.4)	27 (19.6)		
Does the CEO know the percentage of absenteeism?				
PMAQ-CEO 1	94 (75.8)	30 (24.2)		0.291
PMAQ-CEO 2	112 (81.2)	26 (18.8)		
B. Quantitative variables	n	Mean (SD)	Meian (ID ramge	p-value
Cycle				
Percentage of absenteeism				
PMAQ-CEO 1	93	21.4(11.0)	20.0 (14–28)	0.827
PMAQ-CEO 2	112	21.4 (11.0)	20.0 (14.8–27.3)	
Hours (average per professional)				
PMAQ-CEO 1	113	19.9 (8.22)	20.0 (16 – 20)	0.147
PMAQ-CEO 2	137	21.3 (7.78)	20.0 (20 – 21.4)	
Waiting time for treatment (in days)				
PMAQ-CEO 1	94	108 (141)	30 (7–180)	0.701
PMAQ-CEO 2	111	133 (199)	30 (10–180)	

A: Qualitative variables (frequency distribution and association tests – chi-squared); B: Quantitative variables (central tendency and dispersion measures; nonparametric comparison tests - Mann Whitney U).

## Discussion

After the first years of implementation of orthodontic treatment in SUS through the specialized oral health care network, orthodontics was present in about 13% of the CEO, according to the two external evaluation cycles of the PMAQ-CEO, carried out in 2014 and 2018, respectively. We observed that most CEO with orthodontic care are concentrated in the Southeast and Northeast regions, while the Midwest and North regions have few services implemented, reinforcing the poor distribution of services and human resources, as described by the report of the first cycle of the External Evaluation of the PMAQ-CEO for CEO in general,^14^ which included all of these services (whether or not they offered orthodontic treatment). This also points to a similar trend. According to this report, the analysis of the territorial distribution of CEO showed inequality between and within macroregions in the implementation of these services. The North region had the lowest number of services and the Northeast region, the largest.

This problem has already been discussed in the scientific literature and points to inequity in access to the services offered, which aggravates inequalities and imposes barriers to comprehensive and longitudinal care plans.^12,17-19^ Therefore, situational diagnosis, situational strategic planning, and expansion of these services, considering aspects such as demand, territorial extension, installed capacity, among others, will certainly be fundamental strategies,^12,17^ highlighting the importance of government programs such as PMAQ-CEO.

As for human resources, we observed that most professionals were specialists or had MSc or PhD degrees; were civil servants; had been hired by the direct administration; but most had been hired via public tender. There was a statistically significant difference in some aspects, such as: a) Fewer professionals with only refresher courses; b) Fewer professionals hired by other mechanisms (rather than by the direct administration); c) Fewer “other” hiring mechanisms (rather than public competitive examinations and public selection processes) in the second cycle (2018). The observed data reveal, in general, a high level of professional qualification required by the CEO and strictness in the hiring processes; however, the literature indicates a lack of uniformity in these processes, with less rigorous processes in smaller and more economically underprivileged municipalities.^20^

In relation to the work process and inclusion of CEO in the health care network, we observed a significant association between the two external evaluations of the PMAQ-CEO for some outcome variables. As positive aspects of the second cycle (2018), we observed: a) a larger number of services that used single medical records for all specialties (or single records with some specificities); b) more services that used electronic medical records; c) larger presence of monitoring and analysis of goals; and d) greater knowledge about the monthly average of absenteeism in services. As for the positive aspects of the first cycle (2014), we observed a larger number of services related to referrals from primary health care centers.

In general, we noticed that the work process was better in the group evaluated in the second cycle (2018). However, it was already expected that there would be a greater presence of single and electronic medical records, given that using this type of tool was only possible in 2013.^21^ Regarding the inclusion of CEO in the health care network, it is worrying that the largest number of services with demand by referral was evaluated in 2014, because structured services are expected to have greater coordination between the levels of care, with well-defined and agreed-upon protocols for referral and counter-referral. We also observed that there was great variability in the waiting time for treatment in both groups. These problems need to be addressed, as they can negatively interfere in the flow of users of the oral health care network and have consequences such as higher stifled demand or ignorance of the real needs for specialized treatments.

As for absenteeism, there was no statistically significant difference between the groups, but we identified that the measures of central tendency in the evaluated services (in 2014 and 2018) are around 20% for the monthly average of all CEO specialties. However, microdata from the PMAQ CEO external evaluation show that orthodontics is not among the three specialties with the highest absenteeism rates in 107 (86.3%) out of the 124 evaluated CEO,^23^ which may indicate high appreciation of the specialty by the users. The literature points out that treatment by a different professional was the main reason for missed appointments.^23^Given this appreciation, some possibilities regarding the orthodontist’s performance need to be discussed.

It is worth mentioning that there may be a bond between professionals and users because, in addition to this appreciation, orthodontics includes long-term treatments, with periodic appointments. Orthodontists could be a reference of health care for the users, especially adolescents or young adults, and this could be a window of opportunity for raising the awareness of these professionals of the importance of health education for their clinical routine. The literature reports that the reference in health for young women is the gynecologist,^24^ while there is certainly no such reference for men.^25^ Bearing in mind that, in general, women take more care of their health than men do, we believe the establishment of a professional-patient bond can help these users develop this habit.

In addition, even though Brazil joined the select group of countries with a low prevalence of caries at age 12 after the implementation of the Smiling Brazil program, these data are not sustained for adolescents and young adults.^6^ Therefore, orthodontics, as an integral part of comprehensive care, has important characteristics to promote health in a broader fashion and should act to overcome problems that go beyond dental care, thereby meeting the needs of public health services. Still regarding these possibilities, orthodontists can be important actors in the early detection of cancerous lesions. Note that today we are experiencing a new phase of oral cancer, with its diagnosis in young people, non-alcoholics, and non-smokers, which is possibly associated with the transmission of HPV through unprotected oral sex.^26^ Other possibility of early diagnosis is that of primary syphilis, which is characterized by a single, painless ulcerative lesion, without purulent secretion, but with high contamination in the region where the infection has occurred.^27,28^

Expanding the view of health professionals and managers on orthodontics as an integral part of comprehensive care can help solve several problems. A strategy with enormous potential to ensure prevention and treatment of malocclusion, expanding and organizing the offer of dental services, is preventive and interceptive orthodontics by primary health care professionals, which can reduce the stifled demand and the number of complex treatments. It is also necessary to put forward continuing education actions, to have permanent education actions as a routine for professionals, and to put that on the political agenda of decision makers.

Orthodontics is not the only solution to all the problems discussed, but it can contribute to solving them, both in the public and private sectors, with great possibilities of a positive impact on health that goes beyond the limits of orthodontics (when understood as an integral part of comprehensive care). For this to be possible, it is necessary to redefine the role of orthodontists, to guarantee regular education actions, and to include the expanded health clinic concept in their training and in their routine. Managers, professors, researchers, and specialists in the area must focus their attention on the possibilities of improving their work with the incorporation of orthodontics into public services, as part of comprehensive care. The implementation of services in SUS that offer orthodontic treatments was a milestone for the expansion of access to health, but it is necessary to go further and propose solutions that reflect the reality of public services, thus guaranteeing equity and integrality, other doctrinal principles of SUS. Despite the enormous prevalence of malocclusion worldwide, orthodontics is not broadly discussed in the literature in the context of universal health systems.

In relation to the limitations of this study, it was difficult to analyze some variables because they refer to the group of professionals at the CEO and not only to orthodontics. Furthermore, some options for answers to the variables were clustered arbitrarily by the data collection instrument, which made other forms of categorization unfeasible. As for the PMAQ-CEO, it is worth mentioning that, on the one hand, the program contributes to making monitoring an important part of the dynamic processes of health planning, as it is not limited to the processes of evaluation by production indicators. On the other hand, its dynamics is not continuous (some indicators are only monitored when evaluations take place). In addition, the financial incentive for the PMAQ is greater for the best evaluated CEO and not for equity, which can increase the difference in the quality of services, reinforcing inequalities.

## Conclusion

The comparison between the services of the two cycles allowed us to make the following considerations. The poor geographic distribution of the analyzed services reinforces inequalities in the provision of health services in Brazil. Human resources in orthodontics seem to have a satisfactory level of professional education and strictness in hiring processes. Most professionals work 20 or 40 hours per week. As for the work process, there was an increase in the use of single and electronic medical records. The waiting time for service at the analyzed CEO varies considerably. Mixed demand has increased and absenteeism averages around 20% in these services. Access to the treatment of malocclusion is appreciated by users, and orthodontics can contribute to forging a bond between users and health services.

Furthermore, data on specialized care services in Brazil that include orthodontics are promising; however, attention to malocclusion can be improved. We encourage state and local departments to also include orthodontics at other levels of health care, as part of the integrative health and with special attention to the training of primary health care professionals, who can carry out important actions to address the problem with the high prevalence of malocclusion (preventive measures, early diagnosis, less complex care, and referral of users with complex needs to other levels of care).
